# Identification of influential invaders in evolutionary populations

**DOI:** 10.1038/s41598-019-43853-9

**Published:** 2019-05-13

**Authors:** Guoli Yang, Tina P. Benko, Matteo Cavaliere, Jincai Huang, Matjaž Perc

**Affiliations:** 1 Science and Technology on Information Systems Engineering Laboratory, National University of Defense Technology, Changsha, 410073 China; 2 Unit 66136, Beijing, 100042 China; 3 Faculty of Natural Sciences and Mathematics, University of Maribor, Koroška cesta 160, SI-2000 Maribor, Slovenia; 4 School of Computing, Mathematics and Digital Technology, Manchester Metropolitan University, Manchester, UK; 5 CAMTP – Center for Applied Mathematics and Theoretical Physics, University of Maribor, Mladinska 3, SI-2000 Maribor, Slovenia; 6 Complexity Science Hub Vienna, Josefstädterstraße 39, A-1080 Vienna, Austria

**Keywords:** Phase transitions and critical phenomena, Complex networks, Evolutionary theory

## Abstract

The identification of the most influential nodes has been a vibrant subject of research across the whole of network science. Here we map this problem to structured evolutionary populations, where strategies and the interaction network are both subject to change over time based on social inheritance. We study cooperative communities, which cheaters can invade because they avoid the cost of contributions that are associated with cooperation. The question that we seek to answer is at which nodes cheaters invade most successfully. We propose the weighted degree decomposition to identify and rank the most influential invaders. More specifically, we distinguish two kinds of ranking based on the weighted degree decomposition. We show that a ranking strategy based on negative-weighted degree allows to successfully identify the most influential invaders in the case of weak selection, while a ranking strategy based on positive-weighted degree performs better when the selection is strong. Our research thus reveals how to identify the most influential invaders based on statistical measures in dynamically evolving cooperative communities.

## Introduction

Group influence usually will make individuals adopt the behaviors held by their neighbours, leading to the propagation of states throughout the network. Models for the dynamics on networks have been studied in a large number of domains, including the propagation of information, idea, innovation, product, meme, failure, rumor through various networks^1–6^. It is interesting to see that even a small fraction of initial spreaders can trigger a large cascade^7,8^, which can be extended and used in the control of outbreak of epidemics^9^, the conduction of advertisements for products^10^, and the protection of important assets in power grids or cyberspace^11^. In order to identify the influential targets, as known as influence maximization problem (IPM), two types of methods are investigated widely: one is based on operation research, and the other is based on topological centrality.

From the perspective of operation research, people aims to find a set of initial spreaders which can achieve the most prevalent propagation collectively. Kempe *et al*.^12^ proposed the first provable approximation guarantees for efficient algorithms and adopted a natural greedy strategy based on sub-modular function^13^ to obtain nearly 63% of the optimal solution for independent cascade model^14^ and linear threshold model^15^. After that, extensive researches^16–20^ have addressed the influence maximization problem in terms of scalability and uncertainty.

From the perspective of topological centrality, Lü *et al*. reviewed the progresses in the identification of vital nodes and described the state of the art in a systematic review^21^. It is known that the influence of a node is highly dependent on the network structure and its location in the network, but how to calculate the real centrality of a node is nontrivial. Despite there are more than 30 methods to characterize the *importance* of a node, there is not an ubiquitous one which can be applied to different kinds of dynamical systems. Generally speaking, four classes of ranking measurements are employed, as mentioned in^21^, namely ranking based on local information^22–24^, ranking based on global information^25,26^, ranking based on iterative refinement^27,28^, and ranking based on node removal^29,30^.

Notably, the dynamics of various influence spreading models lie on the stochastic triggering of interactions from one state to another one between connecting individuals, including independent cascade model, linear threshold model, epidemic model (such as SI, SIS, SIR), and voter models^31,32^. However, when the dynamics of network is coupled to the evolution of the population (under the framework of evolutionary game theory, EGT), things dramatically change as many results from optimization theory and centrality measures cannot be used to successfully identify the influential invaders, that reproduce and disappear under natural selection. Evolutionary game theory^33–36^ studies a mathematical framework combining game theory^37^ with evolutionary population characterized by the Darwinian process^38^. The competing strategies are formulated by the principles of game theory, where the fitness of an individual is dependent on the neighbourhood configuration and the game settings. In the framework of Prisoner’s Dilemma on networks, the strategy of defection is optimal for individuals, but the strategy of cooperation is the best option for the whole population. A large number of studies^39–64^ have been carried out to explore the relationship between population composition and network structure - these works have shown that the promotion of cooperation is rooted in the formation of highly connected cooperative communities (for reviews see^65–68^).

Few cheaters added into a cooperative community (just like the initial spreaders in propagation networks) will avoid the cost of contributing to the community and can occasionally spread leading to the complete collapse of cooperation and the fragmentation of the community^69^. How to identify the most influential spreaders has been widely investigated in static networks, but the problem remains open for dynamical networks, where the topology of the networks change because of node replications and/or removal^70–72^. In general, the interplay of network and evolutionary dynamics makes challenging the identification of the most influential spreaders in evolving communities. This paper, for the first time to our knowledge, explores the scenarios of the cooperation/cheating conflict in dynamical networks^69^ and proposes an original methodology for the identification of the most influential cheating mutants, i.e., those that can lead to the collapse of cooperation. In the dynamical network model considered in this paper, an initial small fraction of nodes are selected to hold the strategy of defection and compete with the rest of cooperators. As in the case of ranking strategies, we propose and evaluate a weighted degree decomposition (WDD) method to identify the influential nodes which are those associated to the largest possibilities of invasion. The results obtained illustrate that a ranking strategy based on negative-weighted degree can successfully identify the most influential invaders in the case of weak selection, while a ranking strategy based on positive-weighted degree performs better when selection is strong.

## Results

### Model descriptions

We consider a dynamical network with a fixed number of nodes but with a non-fixed number of links which can change during the evolution of the system^69^. Each agent in the network adopts one of the two strategies of the Prisoner’s Dilemma (PD). A cooperator (*C*) pays a cost *c* to provide a benefit *b* to each of its neighbours, where *b* > *c* > 0; however, the cheaters (or defectors, *D*) pay no cost and distribute no benefit. Specifically, if a cooperator has *m* cooperative neighbours and *n* defective neighbours, its payoff is *m*(*b* − *c*) − *nc*. However, a cheater in the same neighbourhood has payoff *mb*. The payoff matrix can be represented by:

1 $$\begin{array}{c}\,\,\,\,C\,\,\,D\\ {\rm{\Pi }}=\begin{array}{c}C\\ D\end{array}(\begin{array}{cc}b-c & -c\\ b & 0\end{array})\end{array}$$

**Figure 1:**
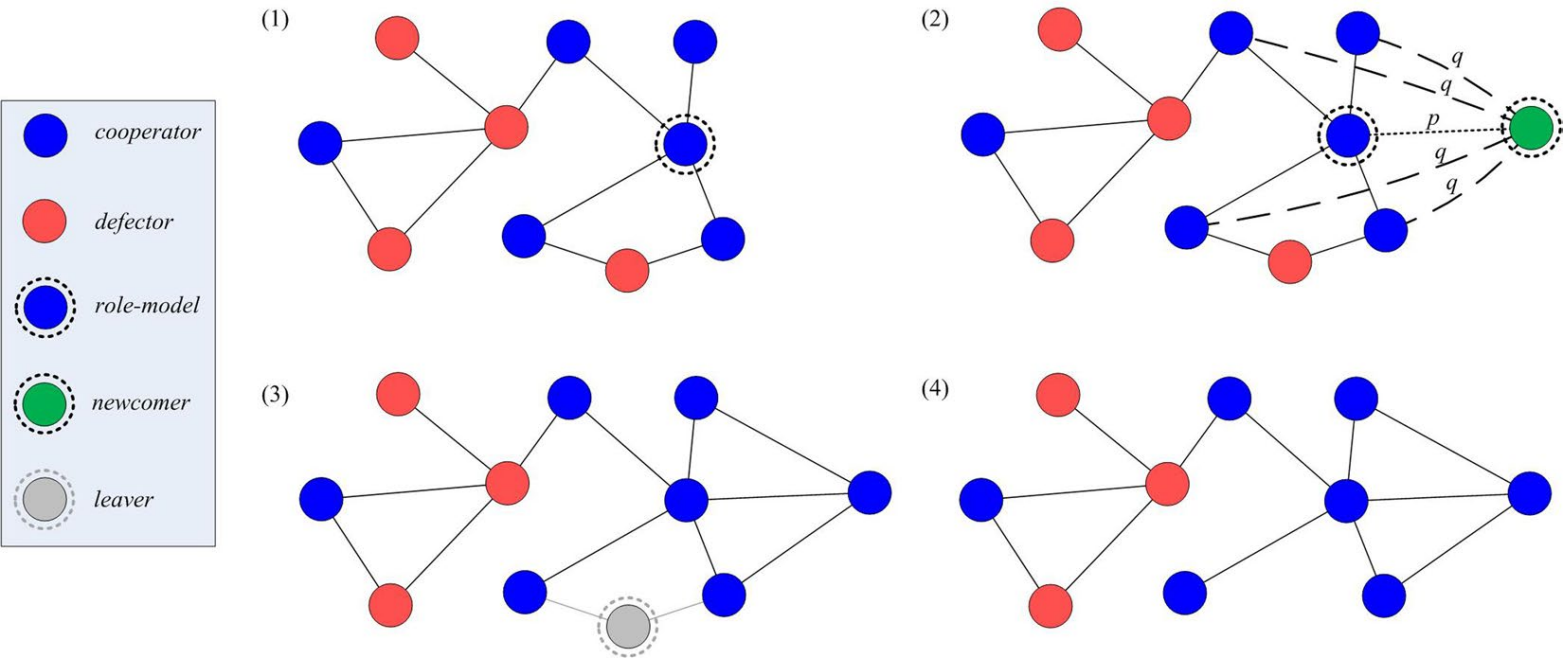
Co-evolution in a structured population. Each update step of the network follows these actions. (1) A role-model is selected to reproduce proportional to its effective payoff. (2) The newcomer connects to the role-model with probability *p* (dashed line), connects to each of its neighbours with probability *q* (dashed lines). *p* and *q* are called embedding parameters. (3–4) A randomly selected node and all its connections are removed from the network.

At each update step, a node *i* is selected as a role-model to reproduce with a probability proportional to its effective payoff, namely the fitness *f*_*i*_:

2 $${f}_{i}={(1+\delta )}^{{\pi }_{i}}$$

where *δ* ≥ 0 specifies the strength of selection and *π*_*i*_ is the sum of payoffs through pairwise interactions to each neighbour. For *δ* = 0 the selection probability is the same for all nodes, while increasing *δ* makes it more likely that a node with a higher payoff is selected to reproduce. In addition, a randomly chosen existing node is removed from the population, so that the number of nodes is constant. The evolutionary dynamics^73^ for a system can be defined by a discrete sequence of update steps as shown in Fig. 1. The dynamics of the strategies can be depicted by differential equations, which are provided in the part of Methods.

We assume that the newcomer adopts the strategy of the chosen role-model (i.e., no mutation occurs), so the composition of population will reach a stationary state once we have [*C*] = *N* or [*D*] = *N*. The parameters *p* and *q* are called embedding parameters, as they explicitly determine the ability of the newcomer to copy the role-model’s social network, which means that the structure of network will always change even when the composition of population is steady.

**Figure 2:**
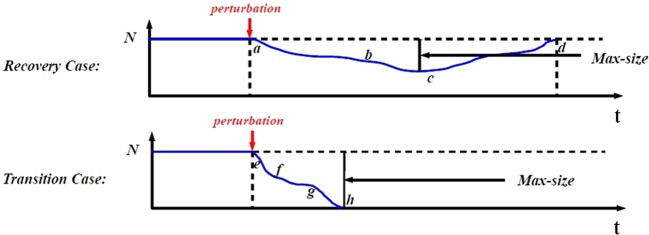
Perturbations lead to recoveries and transitions^69^. We show the two possible outcomes from a perturbation obtained by adding a single cheater in a network of only cooperators. The first row shows the typical case of an unsuccessful perturbation where cooperators resist, while the bottom row shows the typical case of a successful perturbation where cheaters invade gradually. The max-size of invasion is the maximal number of cheaters recorded during a perturbation.

Starting from an ER random network with all cooperators, we run the model for a large number of steps to remove transients and to establish a stable network topology. We then perturb the network by introducing few cheaters and the system is updated until one of the two outcomes is reached: either a case of recovery, the cheaters fail to invade and the system returns to the original state, or a case of transition, the cheaters invade successfully and the original state collapses^69^. Starting from a cooperative community, initially invaded by a single cheater, we illustrate the two possible outcomes in Fig. 2.

### Network configurations

**Figure 3:**
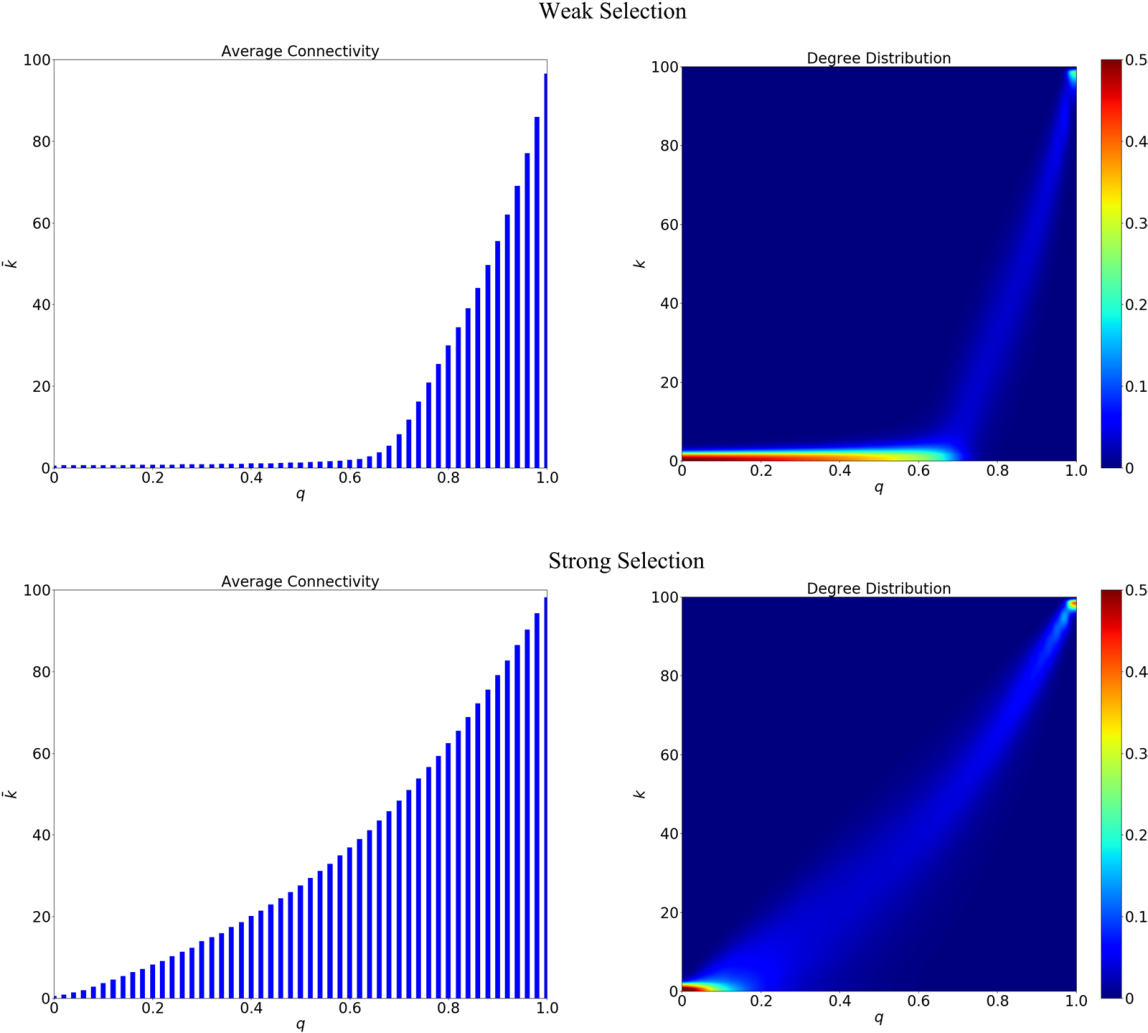
Network connectivity and degree distribution before the invasion of cheaters at weak selection (*δ* = 0.01) and strong selection (*δ* = 0.1) when *q* is changing from 0 to 1. For smaller *q*s, the network gets denser and more diverse at strong selection than that at weak selection due to the emergence of a few *hub* nodes, who are more and more likely to be selected to reproduce.

With a fixed *p* = 0.6, we vary the parameter *q* ∈ [0, 1] to observe the collapse of cooperation and the max-size of invasion. The PD game parameters are *b* = 10 and *c* = *b*/3. Starting from an ER random network (*N* = 100) with average degree of 4, the network reaches stationary state before the introduction of invaders. 10000 independent and identical simulations are run for each *q* for a given size of invaders *n*. The network connectivity and degree diversity before cheaters’ perturbation are illustrated in Fig. 3, where weak selection with *δ* = 0.01 and strong selection with *δ* = 0.1 are considered.

At strong selection with a larger *δ*, a node with a large amount of neighbours (i.e., a large payoff) at the initial stage will gain a larger fitness than that at weak selection (smaller *δ*) as ${f}_{i}={(1+\delta )}^{{\pi }_{i}}$ (See the more details in the Methods). In this way, a node with very high degree will be more likely to be selected as the role-model at strong selection, such that more and more newcomers will be connected to it (as *p* = 0.6) even when the embedding parameter *q* is small. This phenomenon is the so-called *the rich get richer*. That is why we can find the relatively bigger average connectivity and degree diversity for small *q*s at strong selection. When the parameter *q* gets bigger, the network tends to be denser and less diverse for both weak and strong selections.

### Ranking strategies

We investigate several ways (*ranking strategies*) of considering *n* invading cheaters in the network and individuate the strategies which can facilitate the collapse of cooperation.

The initial *n* cheater invaders are placed in a set called *Top-n-set*. This set of selected invaders is constructed in the following way. At the beginning, we have an empty *Top-n-set*, and will insert nodes into it one by one until the size of *Top-n-set* reaches *n*. The nodes inserted in the *Top-n-set* are those ones selected as initial cheaters (invaders), and also called seed nodes.

In order to identify the ranking strategy which will lead to the most influential invaders, we compared the weighted degree decomposition with three classical and standard ranking methods (random ranking, max-degree ranking, and betweenness ranking). For random ranking, *n* random nodes in the network are selected to be the initial cheaters. For max-degree ranking, the *top-n* nodes are selected according to the ranking of their degree. For betweenness ranking, the *top-n* nodes are selected according to the ranking of their betweenness. For the ranking based on *weighted degree decomposition* (WDD), the weighted degree of a node is calculated by:

3 $${k}^{\ast }(v)={k}_{p}(v)+{{\rm{\Sigma }}}_{u\in NS(v)}(\alpha +\beta |NS(u)|)$$

where *k*_*p*_(*v*) is the number of potential nodes in the neighbourhood, and *NS*(*v*) is the set of neighbours who have been selected into the *Top-n-set* as seed nodes (see more details in the Methods). Specifically, two cases are considered:

- positive-weighted degree decomposition: the seed neighbours (i.e. the neighbours who are selected as seed nodes) as well as their seed neighbours are assigned positive weights in the calculation of weighted degree, so that the nodes close to the high-degree clusters are going to be selected. In this way, the nodes with high-degree will not be missed, but the invaders are close to each other. Specifically, we implement the simulations with *α* = 1 and *β* = 0.1.
- negative-weighted degree decomposition: the seed neighbours as well as their seed neighbours are assigned negative weights in the calculation of weighted degree, so that the nodes far away will be selected to avoid the overlap sphere of influence. In this way, some nodes with low-degree distant from the center will be selected, but some nodes with high-degree will be missed. Specifically, we implement the simulations with *α* = −1 and *β* = −0.1.

We use different ranking strategies to select the initial cheaters in the cooperative community. After that, the population evolves according to the procedures shown in Fig. 1. As in the original network model^69^ the selection of the role-model is still implemented with a probability proportional to the effective payoff (i.e., nodes with higher effective payoff have higher chances to be selected as role-models by the newcomers).

### Weak selection case

**Figure 4:**
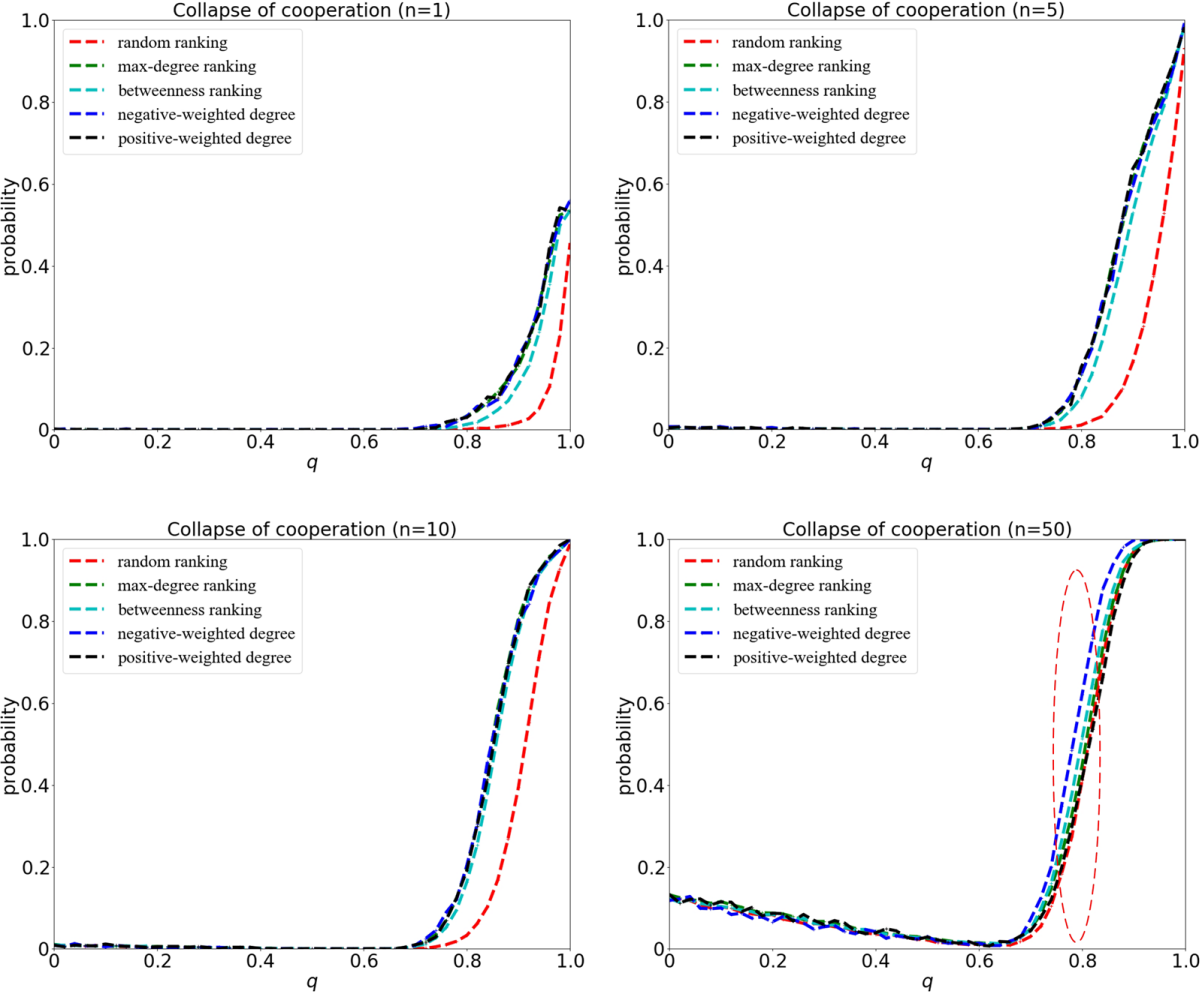
The collapse of cooperation for different ranking strategies at weak selection with *δ* = 0.01. The probability for the collapse of cooperation is computed by the fraction of perturbations that lead to the successful invasion of cheaters out of 10000 perturbation experiments. The x-axis represents the embedding parameter *q* ranging from 0 to 1. The invaders identified by those heuristic and statistical ranking strategies (such as max-degree ranking, betweenness ranking, weighted degree ranking) can lead to a more evident shift of regime than the random one, especially when the value of *n* is not very big. However, the ranking based on negative-weighted degree outperforms others when *n* is large.

At weak selection with *δ* = 0.01, the probability for the collapse of cooperation is illustrated in Fig. 4. Here, four groups of simulations are carried out, which indicate the size of invaders is *n* = 1, *n* = 5, *n* = 10 and *n* = 50, respectively. As we can see, when the size of initial invaders *n* is small, the outcomes of random ranking are distinct from the others. The ranking strategies based on max-degree, betweenness, negative-weighted degree and positive-weighted degree induce a bigger loss of cooperation at bigger *q*s, which reveals the significance of the positions of invaders in dynamically evolving systems. When the size of *n* gets large, however, the differences between each other turn small. In particular, the ranking based on negative-weighted degree outperforms the rest due to the homogeneous network structure and small overlap sphere of influence. Interestingly, this result indicates that *exploration* of far away nodes should be paid more attentions in the case of weak selection.

**Figure 5:**
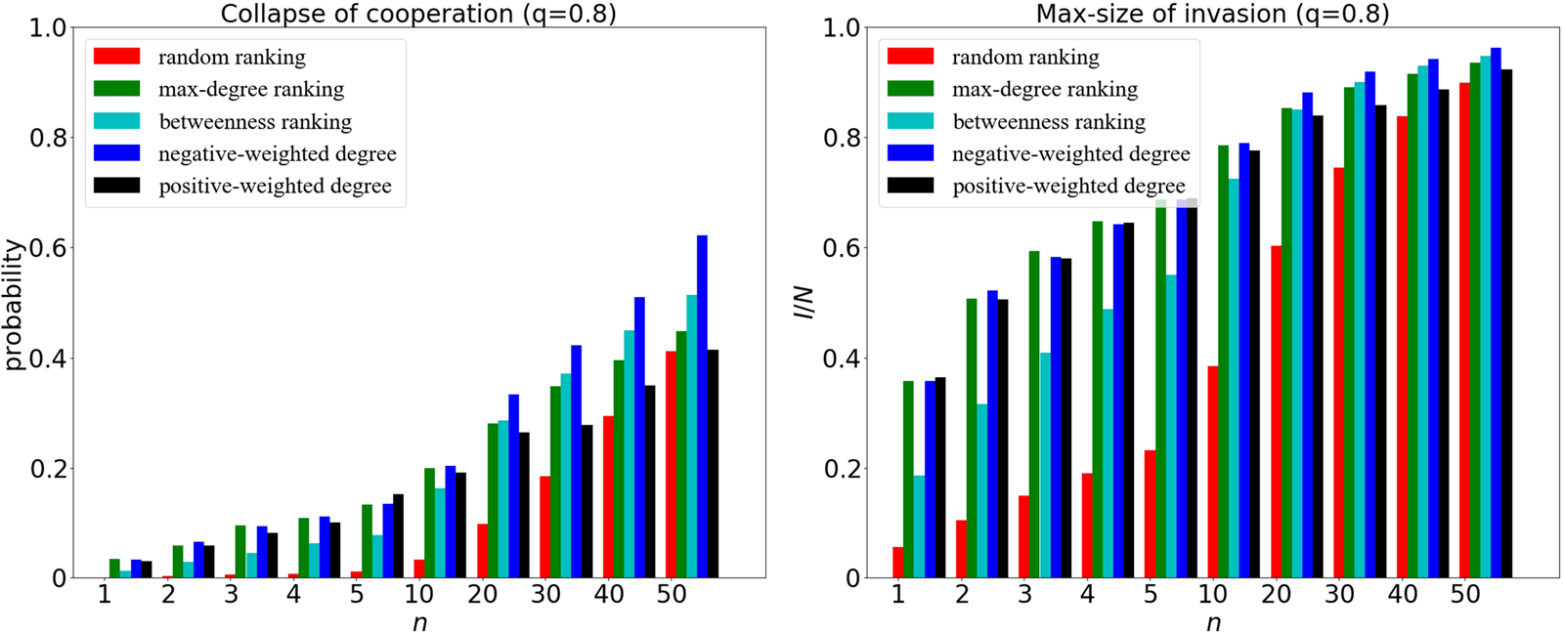
The collapse of cooperation (left panel) and max-size of invasion (right panel) for different ranking strategies at *q* = 0.8 and *δ* = 0.01. The x-axis represents the size of initial cheaters *n*, varying from 1 to 50. Clearly, the ranking strategy based on negative-weighted degree makes the initial invaders distant from each other, leading to a bigger loss of cooperation, especially when *n* is large.

For a given *q* = 0.8, the embedding level of the newcomer agent is high, and we have the collapse of cooperation and max-size of invasion at weak selection in Fig. 5. As we can see, when the size of invaders *n* gets large enough, the ranking strategy based on negative-weighted degree can outperform other ranking strategies in the collapse of cooperation.

**Figure 6:**
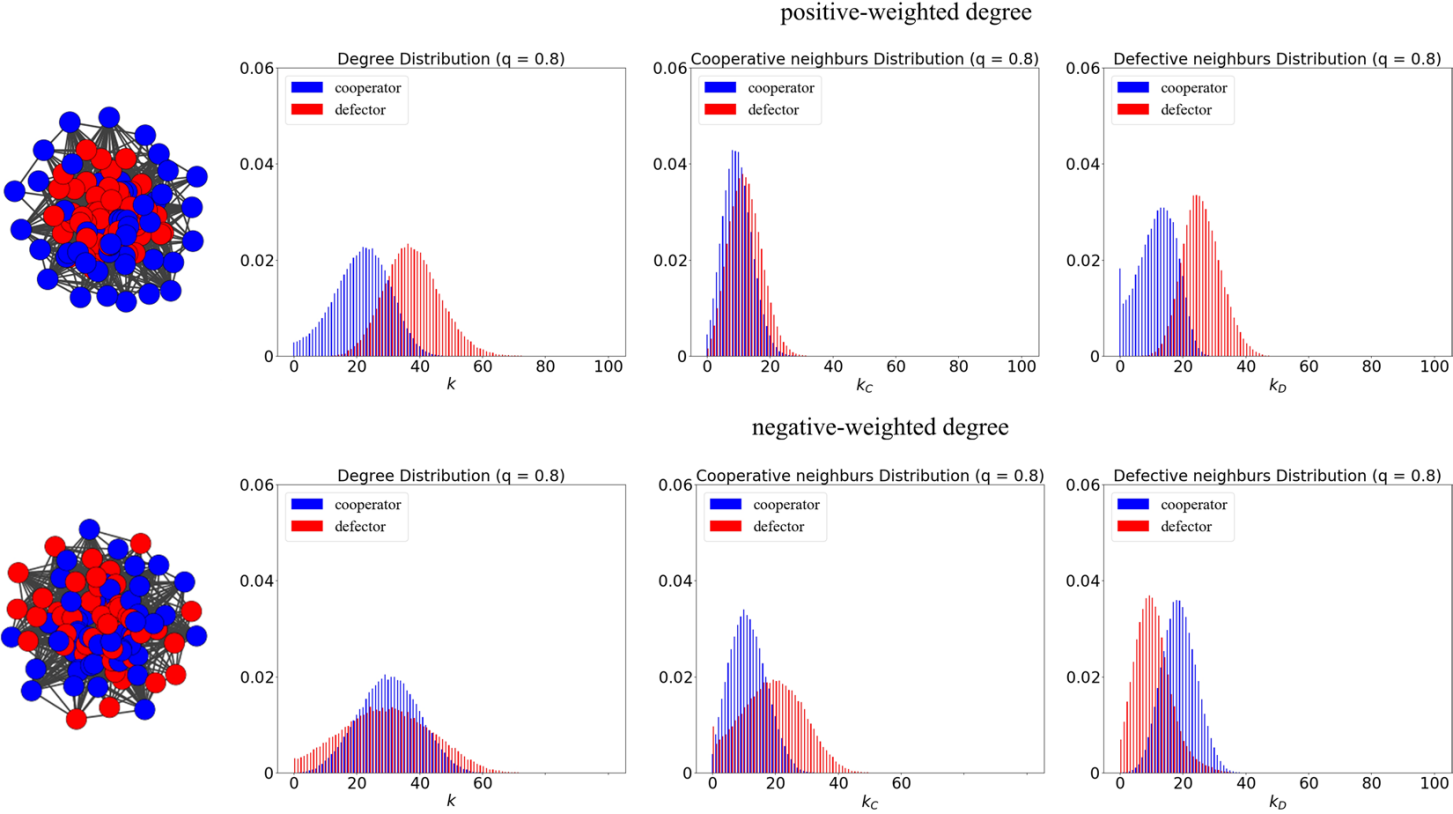
The degree distributions for cooperators and defectors at weak selection (*δ* = 0.01) and high embedding (*q* = 0.8) based on positive-weighted degree ranking (upper panels) and negative-weighted degree ranking (lower panels). Two snapshots are obtained after the invasion of *n* = 50 cheaters. The left panels illustrate the distributions of degree (*k*) for cooperators (blue histograms) and defectors (red histograms), and the middle panels present the distributions of the sizes of cooperative neighbours (*k*_*C*_), and the right panels show the distributions of the sizes of defective neighbours (*k*_*D*_).

To take a deep insight into the difference caused by positive-weighted degree ranking and negative-weighted degree ranking, we obtained the degree distributions for cooperators and defectors at weak selection in Fig. 6. As we can see, a big distinction of degree distributions (*k*) for cooperators and defectors is present under the ranking of positive-weighted degree, while the degree distributions for cooperators and defectors are overlapping under the ranking of negative-weighted degree. As for the distributions of the size of cooperative neighbours (*k*_*C*_), we can find a manifest difference under the ranking of negative-weighted degree, where the defectors hold more cooperative neighbours than the cooperators. On the contrary, for the distributions of the size of defective neighbours (*k*_*D*_), the cooperators hold more defective neighbours than the defectors. As a consequence, the high-degree defectors are distributed over the network, which is dense and well-connected, leading to a bigger loss of cooperation under negative-weighted degree ranking. The snapshots illustrate that the defectors (red nodes) under the ranking of positive-weighted degree tend to be located in the *core*, while the defectors are more likely to keep distant from each other under the ranking of negative-weighted degree.

### Strong selection case

**Figure 7:**
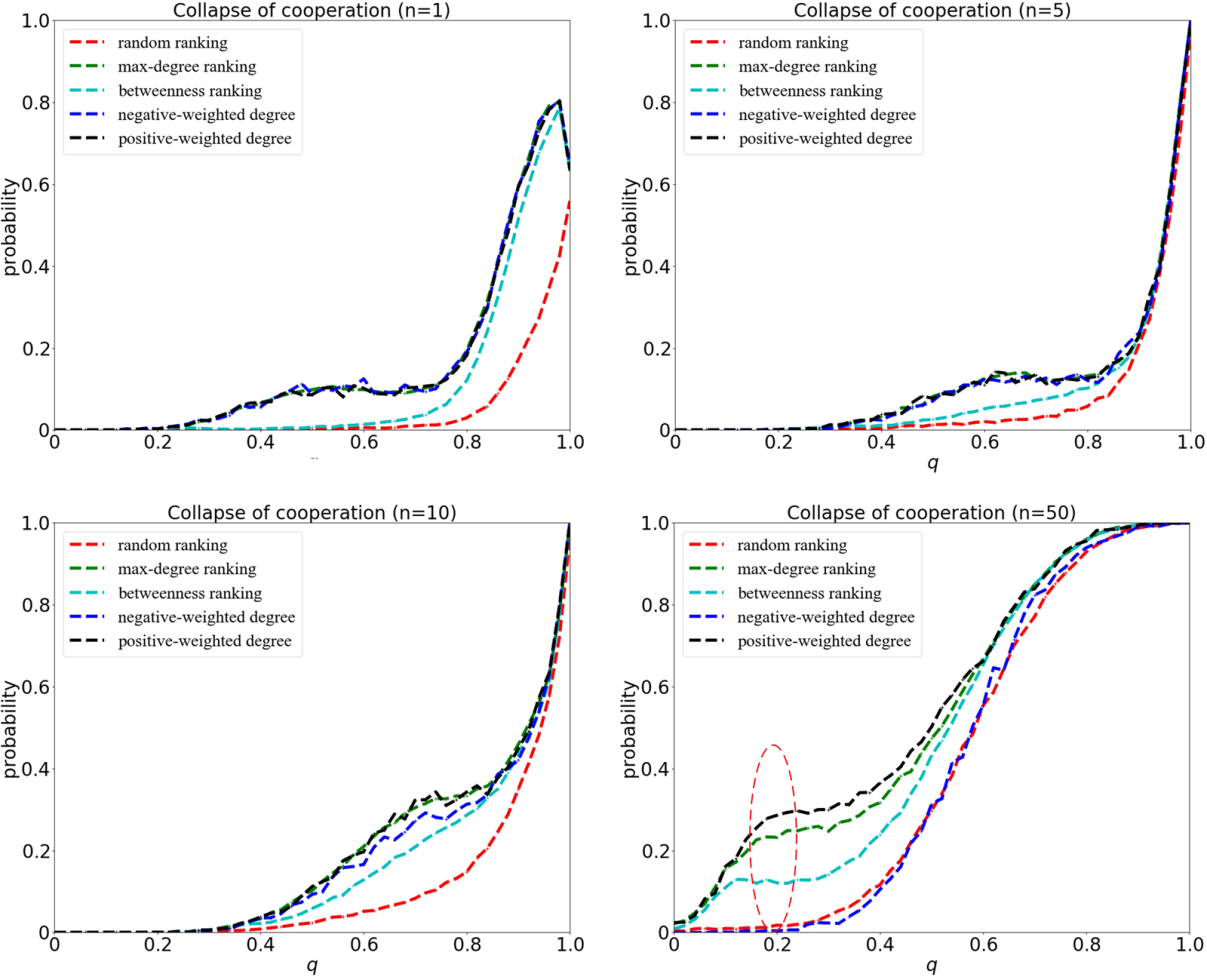
The collapse of cooperation for different ranking strategies at strong selection with *δ* = 0.1. The x-axis represents the embedding parameter *q*, and the y-axis represents the probability for the collapse of cooperation. As we can see, a bigger loss of cooperation is obtained at strong selection even when the embedding parameter *q* is not large. The ranking based on positive-weighted degree works better than the others due to the formation of a heterogeneous network.

At strong selection with *δ* = 0.1, the probability for the collapse of cooperation is illustrated in Fig. 7, which is far from a linear pattern. As we can see, the collapse of cooperation is enhanced by the increase of selection, especially under the ranking of positive-weighted degree and max-degree. As the network structure is more heterogeneous before the invasion at strong selection (see Fig. 3), a few nodes will gain a quite large number of neighbours, but the degree of others is very small. When the size of initial invaders is sufficiently large, the ranking based on negative-weighted degree tries to avoid the gathering of invaders, which will put some low-degree and even isolated nodes far away from the *core* into the *Top-n-set*. However, the ranking based on positive-weighted degree will pay more attentions to the high-degree nodes close to the *core*, so the defectors located in the *interface* can benefit from the connections to the cooperators.

**Figure 8:**
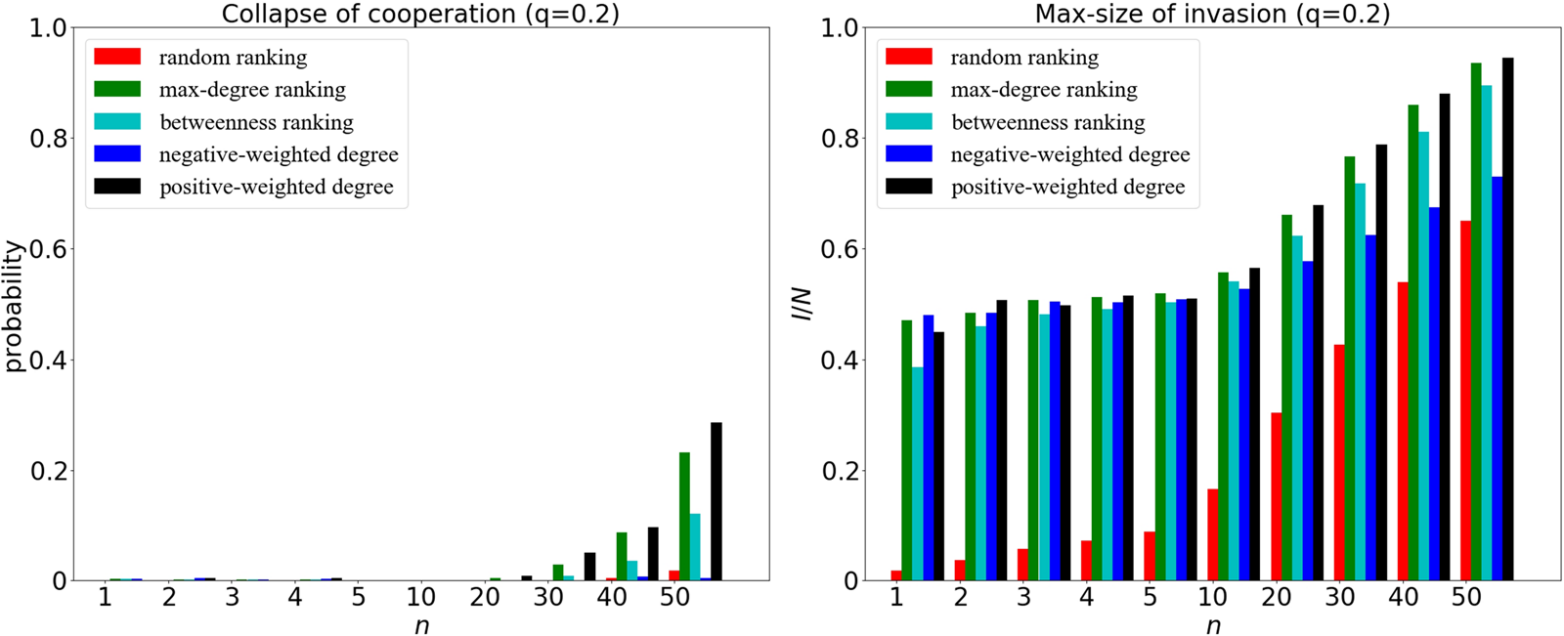
The collapse of cooperation (left panel) and max-size of invasion (right panel) for different ranking strategies at *q* = 0.2 and *δ* = 0.1. The x-axis represents the size of initial cheaters *n*, varying from 1 to 50. Due to the strong selection and low embedding, the ranking strategy based on positive-weighted degree makes the initial invaders close to the *core*, leading to a bigger loss of cooperation especially when *n* is large.

For a given *q* = 0.2, the network connectivity is low as a result of the small embedding level of the newcomer. We can also observe the collapse of cooperation and max-size of invasion at strong selection in Fig. 8. As we can see, when the size of invaders *n* is sufficiently large, the ranking strategy based on positive-weighted degree can outperform the other ranking strategies.

**Figure 9:**
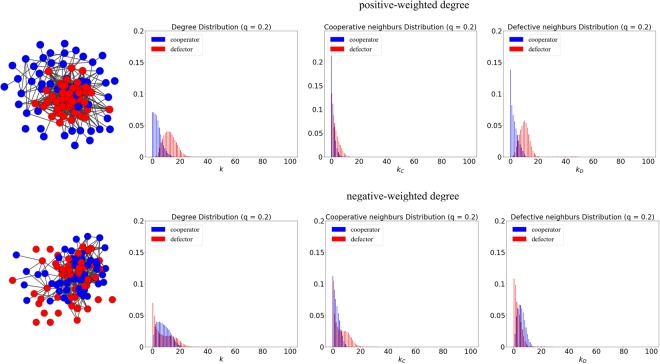
The degree distributions for cooperators and defectors at strong selection (*δ* = 0.1) and low embedding (*q* = 0.2) based on positive-weighted degree ranking (upper panels) and negative-weighted degree ranking (lower panels). Two snapshots are presented after the invasion of *n* = 50 cheaters. The left panels illustrate the distributions of degree (*k*) for cooperators (blue histograms) and defectors (red histograms), and the middle panels present the distributions of the sizes of cooperative neighbours (*k*_*C*_), and the right panels show the distributions of the sizes of defective neighbours (*k*_*D*_).

To take a deep insight into the differences caused by positive-weighted degree ranking and negative-weighted degree ranking, we obtained the degree distributions for cooperators and defectors at strong selection in Fig. 9. As we can see, a big distinction of degree distributions (*k*) for cooperators and defectors is present under the ranking of positive-weighted degree, while the degree distributions for cooperators and defectors are overlapping under the ranking of negative-weighted degree. This indicates that high-degree defectors are well identified through the ranking of positive-weighted degree. As the network connectivity is low and heterogeneity is high due to the small *q* and big *δ*, the *CC* connections are preserved better under the ranking of negative-weighted degree (see the distribution of the sizes of cooperative neighbours), and many defectors with low-degree have no access to the cooperators. On the contrary, the defectors under the ranking of positive-weighted degree have sufficient access to the cooperators, especially for those located in the *interface* between cooperators and defectors. As a consequence, a bigger loss of cooperation under positive-weighted ranking is achieved at strong selection and low embedding. The snapshots illustrate that the cooperators (blue nodes) under the ranking of positive-weighted degree tend to be located on the periphery and maybe even isolated, while the defectors are too far away from the *core* and will more likely to be sparse or isolated under the ranking of negative-weighted degree.

## Discussion

In dynamically evolving structured population, both the positions and the size of the initial invaders are crucial to the stability of the system. In this paper, we have considered the scenario of perturbation invasions where few initial invaders (cheaters) are added to the system (a cooperative community) and selected based on some proposed ranking strategies. As usual, under natural selection, nodes with bigger payoff are more likely to be selected to reproduce, especially when the strength of selection is bigger. In this way, the few initial cheaters can reproduce more often than the cooperators and can ultimately lead to the collapse of cooperation. In this paper weighted degree decomposition (WDD) is proposed as a way to rank the nodes and identify the most influential invaders in the cooperative population.

Interestingly, the ranking strategy based on negative-weighted degree works better at weak selection and for large embedding values. However, the ranking strategy based on positive-weighted degree outperforms the others at strong selection and for low embedding values due to the emergence of heterogeneous networks with low average degrees. This method incorporates the balance between dispersing and gathering into the identification of a set of influential nodes through a general framework, which indicates a trade-off between exploration and exploitation.

To conclude, this work provides a degree discount or reward heuristic for the identification of influential invaders in structured evolutionary populations, which paves a new way to study the influence maximization for dynamically evolving systems in the fields of biology, ecology and sociology. Despite this area is still in its infancy we expect to be a very promising future research line.

## Methods

### Analytical calculation

Let’s derive the dynamics of the evolutionary population by approximate master equations (AME)^31^, which are investigated to construct a system of closed star-like motifs. To capture the dynamics of the stars, we need to consider the following 8 types of dynamics, which are demonstrated through a motif *C*_*m*,*n*_ (a cooperator with *m* cooperative neighbours and *n* defective neighbours).

- (a) the removal of *C*_*m*,*n*_, leading to the decrease of *C*_*m*,*n*_;
- (b) the removal of a neighbour *C* or *D* from *C*_*m*,*n*_, leading to the decrease of *C*_*m*,*n*_;
- (c) the removal of a neighbour *C* from *C*_*m*+1,*n*_ or a neighbour *D* from *C*_*m*,*n*+1_, leading to the increase of *C*_*m*,*n*_;
- (d) the newcomer of *C*_*m*,*n*_ connecting to it, leading to the decrease of *C*_*m*,*n*_;
- (e) the newcomer of *C*_*m*−1,*n*_ connecting to it, leading to the increase of *C*_*m*,*n*_;
- (f) the newcomer of *C*_*m*,*n*_’s neighbour *C* or *D* connecting to it, leading to the decrease of *C*_*m*,*n*_;
- (g) the newcomer of *C*_*m*−1,*n*_’s neighbour *C* or *C*_*m*,*n*−1_’s neighbour *D* connecting to it, leading to the increase of *C*_*m*,*n*_;
- (h) the newcomer of *C*_*x*,*y*_ (where *x* ≥ *m* − 1, *y* ≥ *n*) connecting to *m* cooperators and *n* defectors, leading to the increase of *C*_*m*,*n*_;

Clearly, we have $\,[C]=\sum _{x}[{C}_{x}]=\sum _{x}\sum _{y}[{C}_{x,y}]$, where *C*_*x*_ means the set of cooperators who have *x* cooperative neighbours and [*C*] is the total number of cooperators. Similarly, we have $\,[D]=\sum _{x}[{D}_{x}]=\sum _{x}\sum _{y}[{D}_{x,y}]$. As for the number of various edges, we can obtain them as follows:

$$\begin{array}{ll}\,[CC]=\sum _{x}\sum _{y}x\times [{C}_{x,y}] & \,[CD]=\sum _{x}\sum _{y}y\times [{C}_{x,y}]\\ \,[DC]=\sum _{x}\sum _{y}x\times [{D}_{x,y}] & \,[DD]=\sum _{x}\sum _{y}y\times [{D}_{x,y}]\\ \,[CCC]=\sum _{x}\sum _{y}x\times (x-1)\times [{C}_{x,y}] & \,[CCD]=\sum _{x}\sum _{y}x\times y\times [{C}_{x,y}]\\ \,[CDC]=\sum _{x}\sum _{y}x\times (x-1)\times [{D}_{x,y}] & \,[CDD]=\sum _{x}\sum _{y}x\times y\times [{D}_{x,y}]\\ \,[DCC]=\sum _{x}\sum _{y}x\times y\times [{C}_{x,y}] & \,[DCD]=\sum _{x}\sum _{y}y\times (y-1)\times [{D}_{x,y}]\\ \,[DDC]=\sum _{x}\sum _{y}x\times y\times [{D}_{x,y}] & \,[DDD]=\sum _{x}\sum _{y}y\times (y-1)\times [{D}_{x,y}]\end{array}$$

Here, it is supposed that all neighbours holding same state are indifferent. For a motif *C*_*m*,*n*_, the cooperative neighbour can be approximated by the configuration *C*_1+[*CCC*]/[*CC*],[*CCD*]/[*CC*]_, and the defective neighbour can be approximated by *D*_1+[*CDC*]/[*CD*],[*CDD*]/[*CD*]_. Similarly, for a motif *D*_*m*,*n*_, the cooperative neighbour can be approximated by the configuration *C*_[*DCC*]/[*DC*], 1+[*DCD*]/[*DC*]_, and the defective neighbour can be approximated by *D*_[*DDC*]/[*DD*],1+[*DDD*]/[*DD*]_. When *δ* → 0, we have (1 + *δ*)^*π*^ ≈ 1 + *δπ*, so the sum of fitness can be calculated by:

4 $${\rm{\Sigma }}=\sum _{i}\,{f}_{i}=\sum _{i}\,{(1+\delta )}^{{\pi }_{i}}\approx \sum _{i}\,1+\delta {\pi }_{i}=N+\delta ([CC]+[CD])(b-c)$$

Therefore, the dynamics for the motif *C*_*m,n*_ can be depicted by the following equation:

$$\begin{array}{rcl}\frac{{\rm{d}}}{{\rm{dt}}}[{C}_{m,n}] & = & -1/N[{C}_{m,n}]-(m+n)/N[{C}_{m,n}]+(m+1)/N[{C}_{m+1,n}]+(n+1)/N[{C}_{m,n+1}]\\  &  & -\,[{C}_{m,n}]\frac{1+\delta \pi ({C}_{m,n})}{{\rm{\Sigma }}}(1-(1-p){(1-q)}^{m+1})+[{C}_{m-1,n}]\frac{1+\delta \pi ({C}_{m-1,n})}{{\rm{\Sigma }}}p\\  &  & -\,m[{C}_{m,n}]\frac{1+\delta \pi (\bar{C})}{{\rm{\Sigma }}}q+(m-1)[{C}_{m-1,n}]\frac{1+\delta \pi (\bar{C})}{{\rm{\Sigma }}}q\\  &  & -\,n[{C}_{m,n}]\frac{1+\delta \pi (\bar{D})}{{\rm{\Sigma }}}q+(n-1)[{C}_{m,n-1}]\frac{1+\delta \pi (\bar{D})}{{\rm{\Sigma }}}q\\  &  & +\,\sum _{x\ge m-1,y\ge n}\,[{C}_{x,y}]\frac{1+\delta \pi ({C}_{x,y})}{{\rm{\Sigma }}}((\begin{array}{c}m\\ x\end{array}){q}^{m}{(1-q)}^{x-m}(1-p)(\begin{array}{c}n\\ y\end{array}){q}^{n}{(1-q)}^{y-n}\\  &  & +\,(\begin{array}{c}m-1\\ x\end{array}){q}^{m-1}{(1-q)}^{x-m+1}p(\begin{array}{c}n\\ y\end{array}){q}^{n}{(1-q)}^{y-n})\end{array}$$

Similarly, we can write the differential equations for *D*_*m,n*_. The above derivations provide some analytical calculations for the dynamics of evolutionary population, by which we can estimate the stationary distributions of cooperators or defectors.

Now, let’s investigate the influence of the strength of selection (*δ*) on the evolution of cooperation. Given a network with all nodes holding the state of cooperation, for *δ*_1_ > *δ*_2_, the probability that node *i* will be selected as the role-model at strong selection (namely *δ*_1_) can be approximated by $\frac{1+{\delta }_{1}{\pi }_{i}}{N+{\delta }_{1}[CC](b-c)}$. Similarly, it will be selected as the role-model at weak selection (*δ*_2_) is around $\frac{1+{\delta }_{2}{\pi }_{i}}{N+{\delta }_{2}[CC](b-c)}$. That node *i* will be more likely to be selected at strong selection if and only if:

5 $$\frac{1+{\delta }_{1}{\pi }_{i}}{N+{\delta }_{1}[CC](b-c)} > \frac{1+{\delta }_{2}{\pi }_{i}}{N+{\delta }_{2}[CC](b-c)}$$

Derive the above formula, we have that:

$$\begin{array}{ccc}{\delta }_{2}[CC](b-c)+{\delta }_{1}N{\pi }_{i} &  >  & {\delta }_{1}[CC](b-c)+{\delta }_{2}N{\pi }_{i}\\ \frac{{\delta }_{2}}{{\delta }_{1}}[CC](b-c)+N{\pi }_{i} &  >  & [CC](b-c)+\frac{{\delta }_{2}}{{\delta }_{1}}N{\pi }_{i}\\ (1-\frac{{\delta }_{2}}{{\delta }_{1}})N{\pi }_{i} &  >  & (1-\frac{{\delta }_{2}}{{\delta }_{1}})[CC](b-c)\\ {\pi }_{i} &  >  & [CC](b-c)/N\end{array}$$

As we can see, at the initial state of evolution, a node with a payoff bigger than [*CC*] (*b* − *c*)/*N* will be more likely to be selected as the role-model at stronger selection.

### Weighted degree decomposition

In evolving communities, the spreading of invaders is highly dependent on the large-size size of cooperative neighbours, meanwhile the positions of the invaders shouldn’t be too close together in order to avoid the overlap sphere of influence. Here we will try to propose a new method, namely weighted degree decomposition (WDD), to identify the influential nodes in triggering the shift of regimes in structural population. Just like the method of K-shell^22^ or mixed degree decomposition^24^, we decompose the network gradually according to the weighted degree. The aim of this method is to find a *n*-element set, which will lead to the largest collapse of cooperation.

In general, two types of nodes will be in the network according to the weighted degree decomposition (WDD) method, and they are seed nodes and potential nodes respectively. In detail, we define them by:

- a seed node, who has been selected into the *Top-n-set* as one of the initial invaders;
- a potential node, who is not in the *Top-n-set*, that is to say all the rest are potential nodes to be selected.

Generally speaking, the potential neighbours usually provide positive feedback to the ego agent as they can be used for further spreading. As for the seed neighbours as well as the seed neighbours of seed neighbours, they are usually the nodes holding higher degree and more connections than the ego agent. Two situations need to be considered when we are counting the connections linking the seed nodes: one is *exploitation*, namely taking the links from the seed neighbours into consideration by assigning a positive weight, so that the high-degree seed nodes will get together; and the other is *exploration*, namely discounting the links from the seed neighbours by assigning a negative weight, so that the seed nodes will keep away from each other. In this paper, we compute the weighted degree of a node by the formula:

$${k}^{\ast }(v)={k}_{p}(v)+{\sum }_{u\in NS(v)}\,(\alpha +\beta |NS(u)|)$$

Interestingly, we have two weight coefficients *α* and *β* to make a trade-off between exploitation and exploration. As we can see, once a node has been selected as a seed, it will have a positive or negative influence on its neighbours by changing *α* and *β*. In order to avoid the overlap of sphere of influence, *α* and *β* will set to be negative, and the neighbours’ degree of the seed nodes will be discounted, as a result some remote nodes will be explored. In order to get together to make a strong influence, *α* and *β* will set to be positive, and the neighbours’ degree of the seed nodes will be overrated, as a result the nodes close to the cluster of high-degree seeds will be excessive exploited. It is *α* and *β* that control the weighted degree and the positions of the seeds in the network.

The detailed decomposition is implemented with the following procedure: (1) the node with the maximum weighted degree is selected into the *Top-n-set*; (2) update the states of nodes in the neighbourhood and recalculate the weighted degree for the remaining nodes; (3) repeat the above steps until the size of *Top-n-set* reaches *n*.
